# Supplementary material for: Age-related differences in correction behavior for unintended acceleration
Source: PLoS One. 2020 Jul 10;15(7):e0236053. doi: 10.1371/journal.pone.0236053 (PMC7351190; doi:10.1371/journal.pone.0236053)
Supplement: S1 File — (DOCX) [file pone.0236053.s001.docx]

Hasegawa K, Kimura M, Takeda Y. (submitted). Age-related differences in correction behavior for unintended acceleration.

# Methods

## Questionnaires

To measure the subjective ratings after the experiment, the participants were asked to answer several questionnaires. Self-awareness for cognitive failure in daily life was assessed by the Japanese translated version of the Cognitive Failure Questionnaire (CFQ) ^1,2^. It consisted of 20 questions about a daily failure (e.g., “Do you forget people’s names?”). Participants were required to answer each question using a 5-point scale (1 = *never*, 2 = *seldom*, 3 = *sometimes*, 4 = *frequently*, and 5 = *always*).

Self-awareness of driving behavior on the road was assessed by the Japanese translated version of the Driver Behavior Questionnaire (DBQ) ^3-5^. It consisted of nine questions about errors (e.g., “Do you fail to notice pedestrians crossing?”), violations (e.g., “Do you deliberately disregard the speed limits late at night or very early in the morning?”), and lapses (e.g., “Do you forget where you parked your car?”). As described previously, the participants were required to answer each question using a 5-point scale.

The outcomes of pedal stepping task were assessed by two questions: 1) “Was the current task difficult for you?” (reflects subjective difficulty of the present experiment) and 2) “Do you have much confidence about the performance in the current task?” (reflects the subjective confidence of own performance in the present experiment). Participants were required to answer each question using a visual analog scale of 1-100 (1 = *not at all*, 50 = *neutral*, and 100 = *totally*). Furthermore, only in the older adults, the sense of age-related decline was assessed by a question (“How much do you think to have any ability to execute the current task comparing to the younger adults?”). The older adults were required to answer the question using a visual analog scale of 1–150 (1 = *less than youth*, 100 = *same as youth*, and 150 = *more than youth*).

The daily experiences of pedal misapplication were assessed by two questions that are aimed to find out the anxiety about a pedal misapplication (e.g., “In daily life, how much are you afraid that you will make a pedal misapplication?”) and the near-miss experience caused by pedal misapplications (e.g., “In daily life, how much do you have an experience to nearly make a pedal misapplication?”). Participants were required to answer each question using a 5-point scale (1 = *never*, 2 = *seldom*, 3 = *sometimes*, 4 = *frequently*, 5 = *always*).

# Results

## Questionnaires

The results of questionnaires were shown in Table S1. Independent sample *t*-tests with a factor of age (younger and older) indicated that the violation scores in the Driver Behavior Questionnaire, the self-report about task difficulty, and the self-report about confidence for own task performance significantly differed between the younger and older adults.

**Table S1. The results of the self-reports**

| Questionnaires | Younger | | Older | | Age differences (*df* = 78) | | |
| --- | --- | --- | --- | --- | --- | --- | --- |
|  | *Mean* | *SD* | *Mean* | *SD* | *t* | *p* | *d* |
| CFQ | 2.2 | 0.4 | 2.2 | 0.5 | 0.13 | .898 | 0.03 |
| DBQ (error) | 1.7 | 0.5 | 1.7 | 0.5 | 0.38 | .706 | 0.09 |
| DBQ (violation) | 2.1 | 0.9 | 1.7 | 0.6 | 2.12 | .038 | 0.47 |
| DBQ (lapse) | 1.9 | 0.5 | 2.0 | 0.6 | −0.42 | .678 | −0.09 |
| Difficulty | 46.8 | 21.3 | 63.1 | 25.7 | −2.77 | .007 | −0.62 |
| Confidence | 60.5 | 19.8 | 69.6 | 15.2 | −2.95 | .004 | −0.66 |
| Sense of decline |  |  | 49.9 | 22.1 |  |  |  |
| Anxious | 1.6 | 0.8 | 1.5 | 0.8 | 0.42 | .676 | 0.09 |
| Near-miss experience | 1.3 | 0.6 | 1.4 | 0.6 | −0.95 | .344 | −0.21 |

## Correlations

The correlations between the latencies (in Periods 2 and 3) and the subjective ratings were shown in Table S2. In the younger adults, the self-report of task difficulty positively correlated with the latencies in Period 2. In the older adults, however, no significant correlation was found.

Table S2. The results of Pearson's correlations between the latencies in periods 2 and 3 and the subjective ratings

| Questionnaires | Latencies in Period 2 | | | | Latencies in Period 3 | | | |
| --- | --- | --- | --- | --- | --- | --- | --- | --- |
|  | Younger | | Older | | Younger | | Older | |
|  | *r* | *p* | *r* | *p* | *r* | *p* | *r* | *p* |
| CFQ | .18 | .256 | .16 | .313 | −.03 | .835 | .16 | .309 |
| DBQ (error) | .24 | .136 | .19 | .249 | .08 | .625 | .14 | .386 |
| DBQ (violation) | .28 | .081 | −.04 | .817 | .22 | .178 | −.04 | .796 |
| DBQ (lapse) | .10 | .530 | .30 | .063 | .03 | .870 | .30 | .064 |
| Difficulty | .39 | .013 | −.16 | .339 | .10 | .565 | −.15 | .366 |
| Confidence | −.07 | .670 | −.00 | .992 | −.23 | .145 | .01 | .944 |
| Sense of decline |  |  | .01 | .966 |  |  | −.05 | .763 |
| Anxious | −.16 | .329 | .03 | .856 | −.17 | .283 | .14 | .392 |
| Near-miss experience | .14 | .385 | .04 | .801 | −.09 | .565 | .15 | .352 |

# References

1. Broadbent, D. E., Cooper, P. F., FitzGerald, P. & Parkes, K. R. The Cognitive Failures Questionnaire (CFQ) and its correlates. *Br J Clin Psychol* **21 (Pt 1),** 1–16 (1982).

2. Yamada, N. Shippai keiko shitsumonshi no sakusei oyobi shinraisei datousei no kento [Error proneness questionnaire: Construction, reliability and validity]. *Jap J Edu Psychol* **47,** 501–510 (1999).

3. Reason, J., Manstead, A., Stradling, S., Baxter, J. & Campbell, K. Errors and violations on the roads: A real distinction? *Ergonomics* **33,** 1315–1332 (1990).

4. Komada, Y., Kimura, T., Shinohara, K. & Miura, T. Unten kodo no jikohoukoku ni motoduku unten style no hyoka [The evaluation of driving styles based on the self-report on driving behavior]. *Departmental Bulletin Paper of Osaka University* **34,** 189–214 (2008).

5. Komada, Y., Shinohara, K., Kimura, T. & Miura, T. Unten kodo no jikohoukoku niyoru unten kodo to kodo tokusei no bunrui no kokoromi [The classification of drivers by their self-report driving behavior and accident risk]. *IATSS Review* **32,** 230–237 (2009).
